# Effects of acid on the microstructures and properties of three-dimensional TiO_2_ hierarchical structures by solvothermal method

**DOI:** 10.1186/1556-276X-7-217

**Published:** 2012-04-13

**Authors:** Jing Zhou, Bin Song, Gaoling Zhao, Gaorong Han

**Affiliations:** 1 State Key Laboratory of Silicon Materials and Department of Materials Science and Engineering, Zhejiang University, Hangzhou, 310027, People's Republic of China; 2 Department of Physics, Zhejiang University, Hangzhou, 310027, People's Republic of China; 3, Zhejiang Police College, Hangzhou, 310053, People's Republic of China

**Keywords:** Three-dimensional, TiO_2_, Acid, Solvothermal, Optical properties, Photocatalytic activity, 60, 61.46.Km, 62.23.St

## Abstract

Three-dimensional (3D) TiO_2_ hierarchical structures with various microstructures have been successfully synthesized via a surfactant-free and single-step solvothermal route, in which hydrochloric acid (HCl), nitric acid (HNO_3_), and acetic acid (HAc) are employed as the acid medium, respectively. The effects of acid medium on the microstructures and properties of 3D TiO_2_ hierarchical structure have been studied. The results indicate that 3D dandelion-like microspheres assembled of radial rutile nanorods are obtained in the sample prepared with HCl. Both the fraction of rutile and the diameter of nanorod enhance with the increasing HCl concentration. For the products derived from either HNO_3_ or HAc, 3D spheres composed of anatase nanoparticles are present. The 3D dandelion-like TiO_2_ hierarchical structures show low reflectance and efficient light harvesting since this ordered rod geometry offers a light-transfer path for incident light as well as multiple reflective and scattering effects. Moreover, 3D TiO_2_ with this unique topology shows superior photocatalytic activity despite low surface area, which can be ascribed to the enhanced light harvesting, fast electron transport, and low electron/hole recombination loss.

## Background

Titania (TiO_2_) is widely used in various applications such as photocatalysis of pollutants, catalyst for selective catalytic reduction, photosplitting of water, transparent conducting electrode for dye-sensitized solar cells, electrolyte for proton exchange membrane fuel cells, etc. because of its nontoxicity, low-cost, and chemical stability [1-9]. In the past decades, many efforts have been exerted to the research of nanostructured TiO_2_[10-12]. Numerous studies have revealed that the properties and performance definitely depend on the crystal structures, grain sizes, and morphologies of the TiO_2_ nanostructures [13-15]. Therefore, controlled preparation of TiO_2_ nanostructures is of great scientific and industrial importance. Solvothermal synthesis provides an easy route to prepare a controllable oxide in one step in a tightly closed vessel [16]. TiO_2_ with different structural forms and morphologies has been yielded under diverse solvothermal conditions [17]. Among the various parameters, acid medium and its amount in a solvothermal reaction have been proved to play a substantial role in the control of the morphology and crystal structure of TiO_2_[16,18,19].

On the other hand, it becomes more and more evident that three-dimensional (3D) TiO_2_ assembled with ordered nanostructures under well control may bring some novel and unexpected properties because of its dimension and unique topology [20,21]. For instance, when used as a photocatalyst, 3D microscale TiO_2_ powder is less likely to aggregate and easy to be separated from the solution as compared to nanometer-scale powders [22]. Moreover, the optical properties and photocatalytic activity have been proven to be significantly dependent on the microstructure of 3D TiO_2_. Recently, synthesis of 3D ordered TiO_2_ assemblies of nanoparticles, nanorods, nanowires, and nanotubes have been reported [23-26], for instance, interesting dandelion-like structures [23,26], but the performance and properties are rarely investigated. In our previous work, 3D dandelion-like TiO_2_ structures self-assembled of nanorods have been synthesized in nonpolar solvent based on the water-nonpolar solvent interface via a surfactant-free and single-step solvothermal route [27]. Lately, our subsequent study revealed that the acid medium is essential to the microstructures, optical absorption, diffuse reflectance properties and, consequently, photocatalytic activities of the synthesized 3D TiO_2_ structures. However, the effects of acid medium on the microstructures and properties of 3D TiO_2_ hierarchical structures have not been reported.

Herein, 3D TiO_2_ hierarchical structures with distinct microstructures have been synthesized in various acid media via a surfactant-free and single-step solvothermal route. Hydrochloric acid, nitric acid, and acetic acid were employed in the present systems as the acid medium, respectively. The effects of acid media, including the type and concentration, on the microstructures of TiO_2_ products were studied. The optical absorption and diffuse reflectance as well as photocatalytic performance of 3D TiO_2_ hierarchical structures were investigated.

## Methods

### Synthesis

The synthesis of TiO_2_ samples was based on our previous report with some modifications [27]. All the chemicals were of analytic grade and used without further purification. Titanium *n*-butoxide (TNB) (Ti(OC_4_H_9_)_4_) and *n*-hexane (CH_3_(CH_2_)_4_CH_3_) were used as the titanium precursor and the solvent, respectively. The acid medium introduced in this study includes hydrochloric acid (HCl) (36.5 to 38 wt.%), nitric acid (HNO_3_) (65 to 68 wt.%), and acetic acid (HAc) (CH_3_COOH). In a typical synthesis, 0.45 M TNB and 0.90 M solution of acid were added to *n*-hexane in a total volume of 26 ml. The mixture was loaded into a Teflon-lined autoclave of 50 ml capacity under magnetic stirring and then tightly closed. Subsequently, the autoclave was maintained at 180°C for 4 h followed by natural cooling to room temperature. Afterward, the products were centrifugated and washed with absolute ethanol several times. The final products were dried under vacuum at 80°C for 12 h. The HCl concentration was also tuned from 0 to 1.35 M, keeping all other experimental parameters and procedures unchanged.

### Characterization

The morphologies of TiO_2_ samples were investigated by field emission scanning electron microscopy (FESEM) (S-4800, Hitachi, Ltd., Chiyoda, Tokyo, Japan) and transmission electron microscopy (TEM) (C200, Philips, Germany). The crystal phases of the products were characterized by X-ray diffraction (XRD) (PANalytical X'Pert Pro, Holland, The Netherlands), in a 2*θ* range from 10° to 80°, using Cu K*α* radiation. The microstructure of the product was further analyzed using a high-resolution transmission electron microscopy (HRTEM) (Tecnai G2 F-30, FEI Company, Holland, The Netherlands). The diffuse reflectance was performed on a UV-visible (UV–vis) spectrophotometer (TU-1901, Puxi Analytic Instrument Ltd., Beijing, China) using an integrating sphere with an incident angle of 8°. The optical absorption measurement was calculated by transforming the diffuse reflectance data by the program in apparatus. All the samples for diffuse reflectance measurements are powder samples. The Brunauer-Emmett-Teller (BET) surface areas (*S*_BET_) of the powder samples were determined by nitrogen adsorption-desorption isotherm measurements at 77 K on a Micromeritics TriStar II 3020 nitrogen adsorption apparatus (Micromeritics Instrument Corporation, Norcross, GA, USA). All the samples were degassed at 180°C before the actual measurements.

### Photocatalysis tests

The photocatalytic activity tests were carried out by dispersing TiO_2_ samples in rhodamine B (RhB) solutions and then examining the change of RhB absorbance after UV light irradiation. In a typical procedure, 10 mg TiO_2_ powder was dispersed in 10 mL RhB aqueous solution with an initial concentration of 10^−5^ M. This reaction dispersion was magnetically stirred in the dark for 60 min prior to irradiation to establish the adsorption/desorption equilibrium. The TiO_2_/RhB solution was then exposed to UV light from an 80 W xenon arc lamp with a 420 nm cutoff filter, and the irradiance on the samples is 70 μW cm^−2^. After illumination, the TiO_2_ powder was removed from the suspension by centrifugation before the absorption spectrum was taken by a UV–vis spectrophotometer. The photocatalytic activity could be characterized by an apparent first-order rate constant *k*, which could be calculated using the following equation:

(1) $$k=\frac{\ln\left(\frac{A_{0}}{A}\right)}{t}\text{,}$$

where *A*_0_ is the absorbance of the initial RhB solution at 553 nm, and *A* is the absorbance of RhB at 553 nm when the irradiation time is 5, 10, 15, and 20 min, respectively.

## Results and discussion

### Effects of acid media on the microstructures of TiO_2_

**Figure 1:**
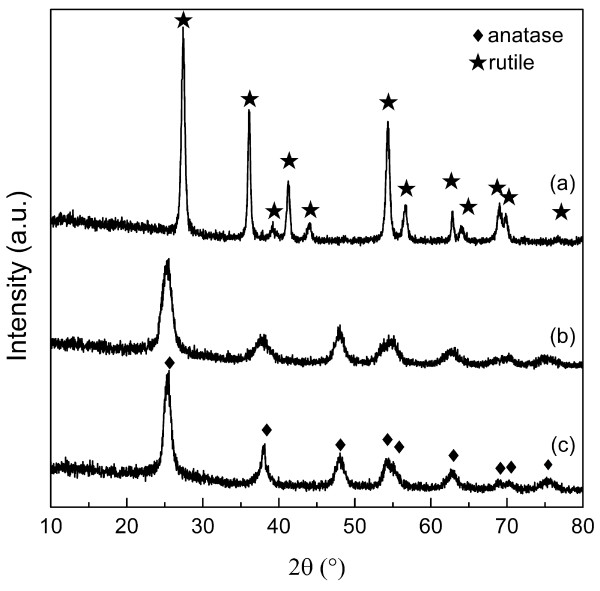
**XRD patterns of the products.** Products synthesized using various acid media at a concentration of 0.90 M: **(a)** HCl, **(b)** HNO_3_, and **(c)** HAc.

Figure 1 shows the XRD patterns of the products synthesized using HCl, HNO_3_, and HAc as the acid medium at a fixed concentration of 0.90 M, respectively. Pure rutile (JCPDS no. 21–1276) is formed in HCl medium, while pure anatase (JCPDS no. 21–1272) is generated in both HNO_3_ and HAc media. The crystal sizes are calculated from either rutile (110) or anatase (101) diffraction using Scherrer's equation [28]. The results show that the estimated crystal sizes of the samples from HCl, HNO_3_, and HAc medium are 15.4, 5.4, and 8.5 nm, respectively.

**Figure 2:**
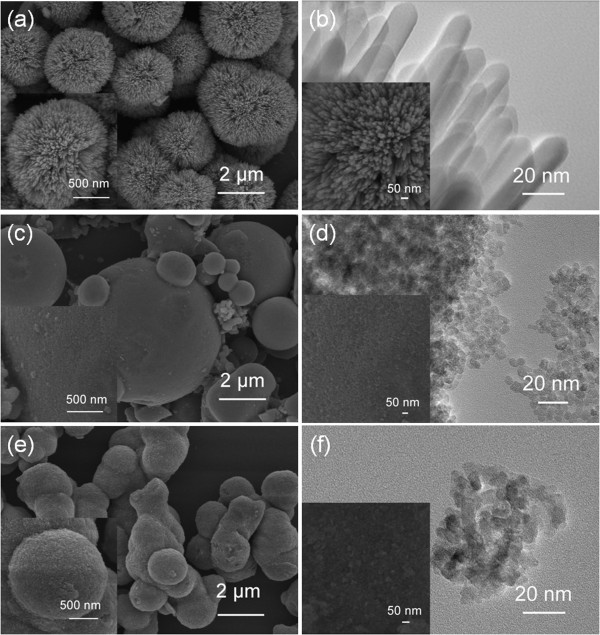
**Typical FESEM and TEM images of the products.** Products synthesized using various acid media at a concentration of 0.90 M: **(a and b)** HCl, **(c and d)** HNO_3_, and **(e and f)** HAc. **(a)**, **(c)**, and **(e)** are overview FESEM images. **(b)**, **(d)**, **(f)** and the insets are enlarged TEM and FESEM images of the surfaces of the corresponding samples.

Figure 2 shows the typical FESEM and TEM images of the products synthesized with various acid media at a concentration of 0.90 M. For the sample prepared with HCl, numerous nanorods are highly aggregated into 3D dandelion-like spheres in mean diameter of 2.1 ± 0.2 cm (*N* = 8, *N* is the number of measurements) (see Figure 2a and inset), forming a hierarchical structure. Figure 2b and inset show the magnified FESEM and TEM images of the nanorods, indicating that the nanorods possess a rectangular shape with an average diameter of 15.1 ± 1.3 nm (*N* = 12) and smooth side wall. For the samples derived from either HNO_3_ or HAc, 3D spheres with a nonuniform size distribution are present (see Figure 2c for HNO_3_ and Figure 2e for HAc). Magnified TEM and FESEM images of the surfaces (see Figure 2d for HNO_3_ and Figure 2f for HAc) show that both the spheres are composed of nanoparticles, and the primary particle sizes are about 5.0 ± 0.5 (*N* = 12) and 8.2 ± 0.9 nm (*N* = 12), respectively.

**Figure 3:**
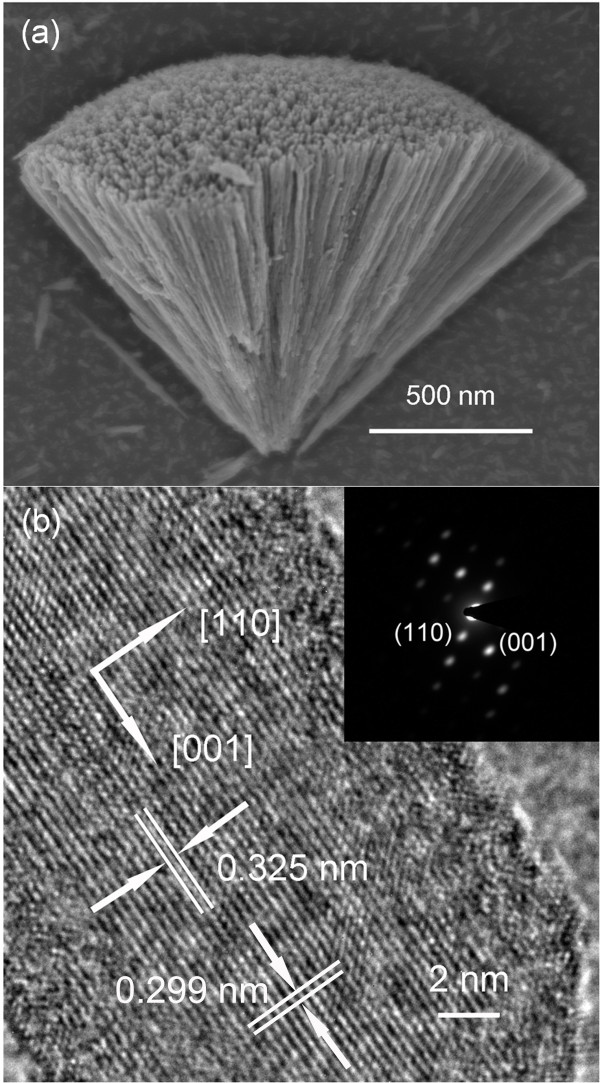
**FESEM and HRTEM images and corresponding SAED pattern.(a)** FESEM image of a sector and **(b)** HRTEM image and corresponding SAED pattern (inset) of an individual nanorod in the product using HCl acid medium at a concentration of 0.90 M.

Further characterization of the 3D dandelion-like structure synthesized in 0.90 M HCl medium is provided by the FESEM and HRTEM images shown in Figure 3. The typical section of a sector from a sphere (Figure 3a) confirms that the microsphere is composed of numerous nanorods of about 1 μm in length, radiating from the center to form a spherical dandelion-like structure. In the HRTEM image (Figure 3b), the distances between the adjacent lattice fringes are measured to be 0.325 and 0.299 nm, which agree well with the interplanar distances of rutile TiO_2_ (110) and (001) (JCPDS no. 21–1276), indicating the growth direction of [001], parallel to *c*-axis. The corresponding selected area electron diffraction (SAED) pattern (inset in Figure 3b) demonstrates that the nanorod is single crystalline and could be indexed to the pure rutile TiO_2_ phase. Combined with the XRD data and HRTEM image, such features imply the single crystal with the exposed surface of (110) crystal plane [29-31].

The results reveal that the precursor containing HCl as the acid medium has a tendency to form rod-shaped rutile titania, whereas anatase particles are obtained when either HNO_3_ or HAc is used. The formation of the two different morphologies and crystal structures depends on the presence of diverse ions (Cl^−^, NO_3_^−^, or CH_3_COOH^−^) during the synthesis. It is known that anatase nucleates and grows first according to Ostwald's law, and rutile nuclei appears eventually when the successive reaction is taking place since rutile is the thermodynamically most stable phase [16]. Thereafter, rutile quickly grows epitaxially at the expense of mother anatase crystallites via a dissolution and precipitation process [32]. This is also the reason why the crystalline sizes of rutile are always greater than anatase, as shown in Figures 1 and 2.

Both rutile and anatase belong to the tetragonal crystal system, consisting of TiO_6_ octahedra as a fundamental structural unit. Their crystalline structures differ in the assembly of the octahedral chains [19,33]. Anatase results from face-shared linking of octahedra, while rutile is built by edge-shared linking [34]. Rutile has 4_2_ screw axes along the crystallographic *c*-axis. The screw structure promotes crystal growth along this direction, resulting in a crystal morphology dominated by the {110} faces [35]. Therefore, rutile nanoparticles are usually rod-like.

The structural rearrangement of octahedral TiO_6_ units is essential during the phase transformation. It has been known that Cl^−^ anions show weaker affinity to a titanium atom in an aqueous solution than NO_3_^−^ and CH3COO^−^ anions do [16]. Therefore, pure rutile phase could be obtained in HCl aqueous solution more easily. On the other hand, *n*-hexane is used as the solvent, which is a nonpolar solvent. The liquid-liquid (nonpolar solvent-water) interface in the present system provides an ideal place for self-assembly of 3D nanostructures [36,37]. In HCl medium, nanorods self-assemble into 3D dandelion-like spheres. Their radial assembly in a good geometrical match to the spherical structure could significantly reduce the total free energy [17]. For both HNO_3_ and HAc, the strong affinity of NO_3_^−^ and CH3COO^−^ anions to titanium inhibits the structural rearrangement and, subsequently, the phase transformation [16]. Accordingly, pure anatase is present in both HNO_3_ and HAc media due to stronger chemical coordination to titanium. Irregular 3D microspheres composed of anatase nanoparticles are obtained in either HNO_3_ or HAc medium (see Figure 2c,d,e,f).

### Effects of hydrochloric acid concentration on the microstructures of TiO_2_

**Figure 4:**
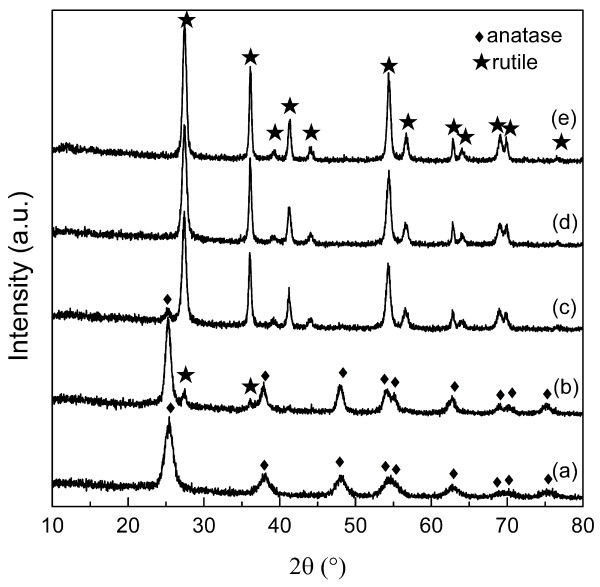
**XRD patterns of the products.** Products synthesized at various HCl concentrations: **(a)** 0, **(b)** 0.45, **(c)** 0.68, **(d)** 1.12, and **(e)** 1.35 M.

**Figure 5:**
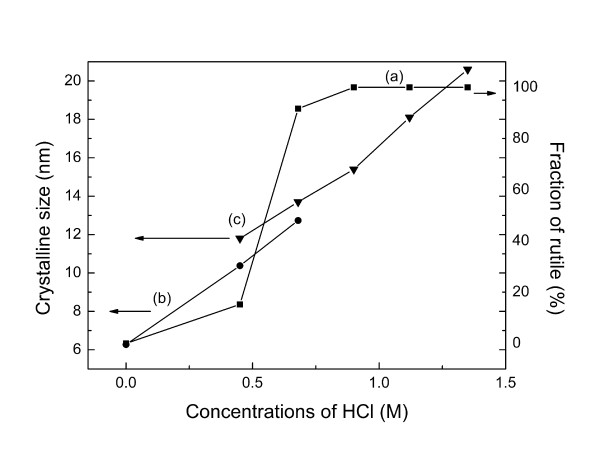
**Influence of HCl concentrations.(a)** The fractions of rutile, **(b)** crystalline sizes of anatase, and **(c)** crystalline sizes of rutile.

According to the above experimental results with inorganic acid media, it can be concluded that 3D spherical structure composed of rutile nanorods can be formed in HCl. Therefore, HCl solution was selected as a representative acid medium to investigate the growth of 3D dandelion-like structure under various acidity conditions. Figure 4 shows XRD patterns of the products synthesized at various HCl concentrations. Pure anatase (JCPDS no. 21–1272) is formed in the sample without addition of any acids (Figure 4a). At an HCl concentration of 0.45 to 0.68 M, anatase (JCPDS no. 21–1272) and rutile (JCPDS no. 21–1276) coexist in the product (Figure4b,c). The intensity of characteristic peaks of rutile becomes stronger with increasing HCl concentration. Pure rutile (JCPDS no. 21–1276) is obtained when HCl concentration is 1.12 to 1.35 M (Figures 1a and 4d,e). The results indicate that higher HCl concentration favors rutile crystallization. The rutile ratio (*χ*_R_) of each sample is estimated from XRD intensity data using the formula expressed as follows:

(2) $$\chi_{\text{R}}={\left[1+\frac{0.8I_{\text{A}}}{I_{R}}\right]}^{-1}\text{,}$$

where *I*_A_ and *I*_R_ are the integrated intensity of anatase (101) and rutile (110) diffraction peaks, respectively [13,38]. The fraction of rutile tends to be greater at higher HCl concentrations (Figure 5a). Pure rutile phase could be ultimately obtained at an HCl concentration of 0.90 M or higher. The concentration of HCl affects not only the fraction of rutile but also the nanocrystal size in the products. The crystal sizes of the TiO_2_ samples were determined from the diffraction peak broadening using the Scherrer formula shown in Figure 5b,c. Larger crystalline sizes of either anatase or rutile are obtained at higher HCl concentrations, which can be confirmed by the FESEM and TEM results.

**Figure 6:**
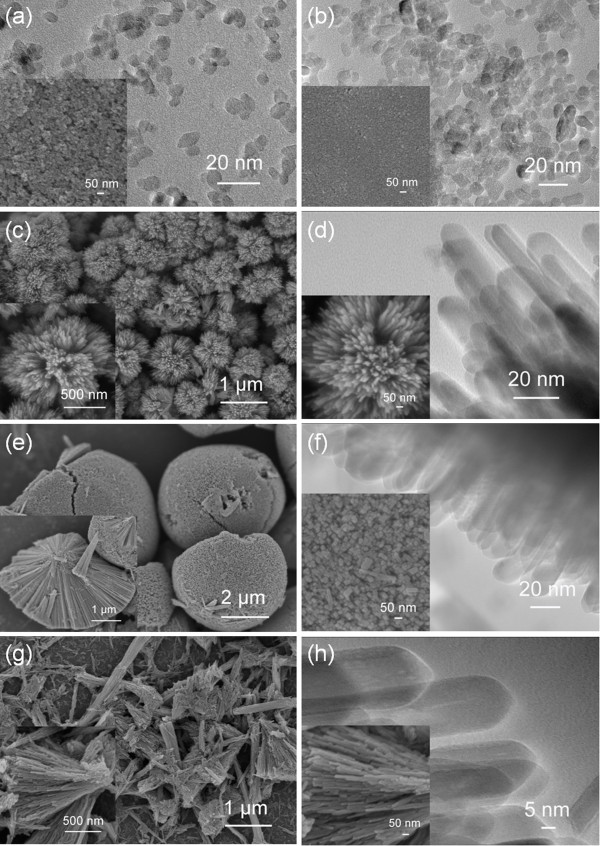
**Typical FESEM and TEM images of the products.** Products synthesized at various HCl concentrations: **(a)** 0, **(b)** 0.45 M, **(c and d)** 0.68 M, **(e and f)** 1.12 M and **(g and h)** 1.35 M. **(c)**, **(e)**, and **(g)** are overview FESEM images. **(d)**, **(f)**, **(h)** and the insets are enlarged TEM and FESEM images of the surfaces of the corresponding samples.

Figure 6 shows the typical FESEM and TEM images of the products synthesized at various HCl concentrations. The samples prepared at HCl concentrations of 0 and 0.45 M assume bulky aggregation of nanoparticles with an average size of 6.1 ± 1.2 (*N* = 12) and 11.4 ± 0.7 nm (*N* = 12) (see Figure 6a,b), respectively. At higher HCl concentration (0.68 or 1.12 M), 3D hierarchical dandelion-like spherical structures generate. The mean diameters of the nanorods are 13.5 ± 1.4 (*N* = 12) and 17.8 ± 1.7 nm (*N* = 8) at HCl concentrations of 0.68 and 1.12 M (Figure 6d,f), respectively. Meanwhile, together with the sample synthesized with 0.90 M HCl (see Figure 2a,b), it can be found that the rod diameter, rod density, and size of spheres gradually enhance with increasing HCl concentration. The 3D spheres become noticeably not so round when the HCl concentration reaches as high as 1.12 M. Further elevating the HCl concentration to 1.35 M, 3D dandelion-like structures are collapsed into sectors composed of nanorods with an average diameter of 19.8 ± 1.8 nm (*N* = 5).

It has also been widely reported that higher acid concentration favors the formation of rutile phase since the transformation from anatase to rutile could also be promoted by strong acidity [13,19,35]. In this study, the fraction of rutile enhances with increasing HCl concentration. Bulky aggregated rounded particles are present in the samples of pure anatase or mainly composed of anatase for the low concentration of HCl. When the HCl concentration is 0.68 or higher, rod-like particles are obtained in the sample mainly composed of rutile or pure rutile. Pure rutile is obtained at the HCl concentration of 0.90 M or higher. Acid can accelerate not only phase transformation but also crystal growth [16]. Crystal growth of both anatase and rutile particles can be facilitated in stronger acid solution, respectively. In the present case, either anatase or rutile nanocrystals tend to be larger at higher HCl concentration. Meanwhile, the rod density in the 3D dandelion-like spheres gradually enhances with increasing HCl concentration. Furthermore, both highly acid condition and selectively adsorption of Cl^−^ on rutile (110) plane accelerate the anisotropic growth along [001] orientation [13,35]. Therefore, 3D dandelion-like structures are destroyed when the HCl concentration reaches as high as 1.35 M, resulting in sectors composed of nanorods.

### Optical properties of 3D TiO_2_ structures

**Figure 7:**
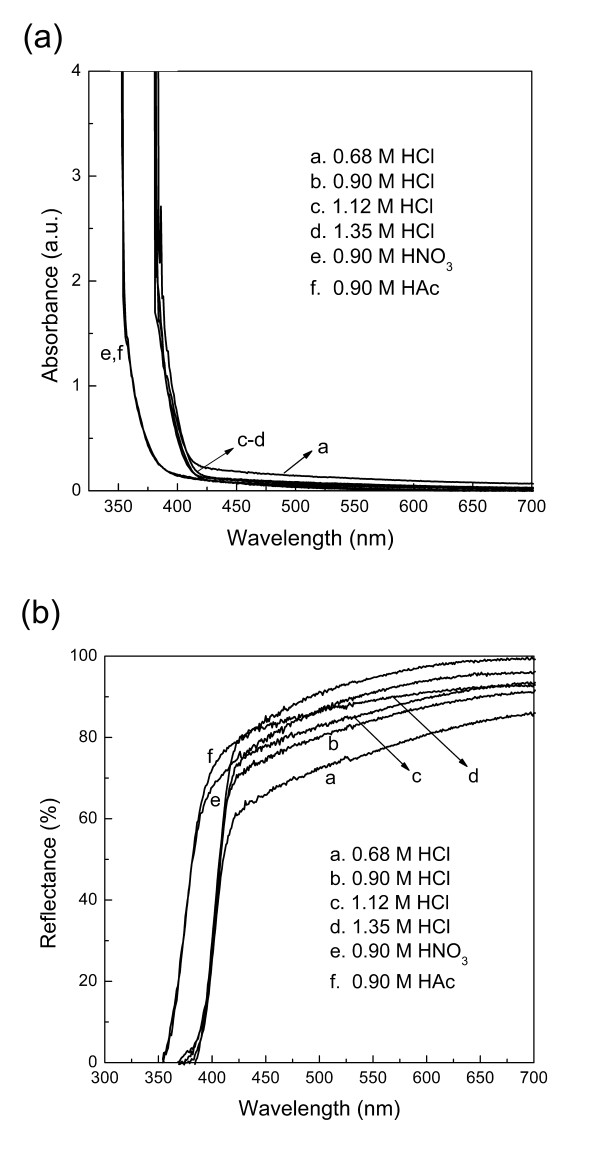
**Optical absorption (a) and diffuse reflectance spectra (b) of 3D TiO**_**2**_**structures.** Structures synthesized with various acid conditions: 0.68 M HCl, 0.90 M HCl, 1.12 M HCl, 1.35 M HCl, 0.90 HNO_3_, and 0.90 M HAc, respectively.

The optical absorption and diffuse reflectance spectra of 3D TiO_2_ structures synthesized with various acid conditions are shown in Figure 7. As seen in Figure 7a, all the samples exhibit low absorption in the visible light range since nanosized TiO_2_ is a visible-light-transparent material. The slight visible light response for the sample prepared with 0.68 M HCl might be due to the presence of defects in the rutile-anatase mixed structures [39]. The abrupt increase below 400 nm was due to the absorption of light caused by the excitation of electrons from the valence band to the conduction band of TiO_2_. The absorption edge wavelength of the samples derived from HCl is shorter than that of the samples prepared with either HNO_3_ or HAc, which can be ascribed to their different crystal phase structures and the narrower band gap of rutile TiO_2_ (3.0 eV) compared with that of anatase (3.2 eV). The diffuse reflectance spectra shown in Figure 7b also show the red shift in the band gap transition to longer wavelengths for samples prepared with HCl. In UV light region (<420 nm), TiO_2_ prepared with 0.68, 0.90, or 1.12 M HCl exhibits lower reflectance than that prepared with either HNO_3_ or HAc. This can be ascribed to the unique 3D dandelion-like structure in which the interspace between ordered rods act as a light-transfer path for introducing incident light into the inner surface of TiO_2_. This allows UV light waves to penetrate deep inside the TiO_2_ structure. The nanorods also offer multiple reflective and scattering effects of UV light, preventing the incident waves from bouncing back to the free space [40,41]. Both of these give rise to a lower reflectance and more efficient light harvesting. Among them, TiO_2_ prepared with 0.68 M HCl shows lowest reflectance since tapered tips are beneficial to lower reflectance in comparison with densely arranged flat tops [41,42]. The increase in reflectance for TiO_2_ prepared with 1.35 M HCl can be due to the collapse of 3D dandelion-like structure [40].

### Photocatalytic activity of 3D TiO_2_ structures

**Figure 8:**
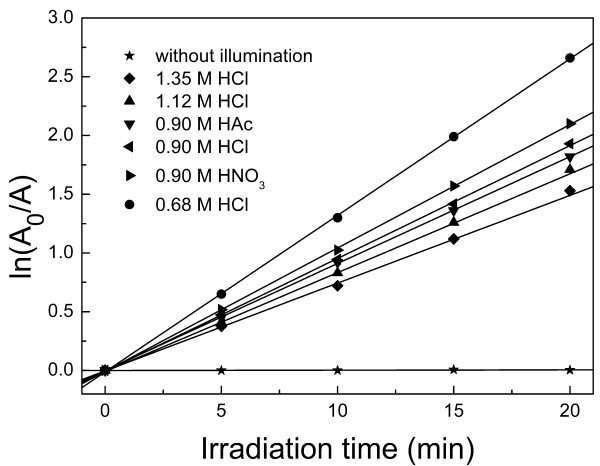
**Plots of photocatalytic degradation of RhB using 3D TiO**_**2**_**structures.** Structures synthesized with various acid conditions: 0.68 M HCl, 0.90 M HCl, 1.12 M HCl, 1.35 M HCl, 0.90 HNO_3_, and 0.90 M HAc, respectively. The plot ‘without illumination’ represents the typical plot of photocatalytic degradation of RhB without illumination of the TiO_2_/RhB solution for TiO_2_ sample prepared with 0.68 M HCl since all the samples show almost the same plot without illumination.

The photocatalytic activity of TiO_2_ samples can be quantitatively evaluated by comparing the rate constants *k*, which is calculated by the plots of photocatalytic degradation of RhB as shown in Figure 8. In order to eliminate the influence of adsorption, the typical plot of photocatalytic degradation of RhB without illumination of the TiO_2_/RhB solution is given in Figure 8. It can be seen that without illumination, the RhB absorptance remains almost unchanged. This assures that the reduction of RhB absorptance comes from the photocatalytic effect of the TiO_2_ samples rather than powder adsorption. Higher value of *k* indicates better photocatalytic activity. The *k* values for the degradation of RhB and BET surface areas are presented in Table 1. It is commonly accepted that mesoporous TiO_2_ with a large surface area is a superior photocatalyst since a larger surface area provides more active adsorption sites. That is why the sample prepared with HNO_3_ shows a slightly greater photocatalytic capacity than that with HAc. However, it is difficult to explain the high activity of 3D dandelion-like TiO_2_ solely based on its surface area. It can be seen that the BET surface area of TiO_2_ synthesized with HCl is much lower than that with HNO_3_ or HAc, while the 3D TiO_2_ prepared with 0.68 or 0.90 M HCl exhibits higher or comparable *k* value compared with that derived from HNO_3_ or HAc. Such higher photocatalytic performance than expected can be ascribed to the unique topologies and optical properties of the 3D dandelion-like TiO_2_. The overall photocatalytic activity is governed by light harvesting efficiency, efficiency of the reaction of photogenerated electron/hole (*e*^−^/h^+^), and the rate of *e*^−^/h^+^ recombination. Firstly, the absorbance is strongly influenced by the topologies of photocatalyst. For 3D dandelion-like TiO_2_, the interspaces between ordered nanorods serve as a light-transfer path, making it possible to illuminate even the core TiO_2_ particles. Considering the improved light absorption, reduced reflectance, and scattering within such a hierarchical system, the effective light-activated surface area can be significantly enhanced. This would improve the photoabsorption efficiency of the photocatalyst. On the other hand, only the surface layer contributes to the light harvesting for spheres composed of nanoparticles. Moreover, the oriented 1D geometry provides a direct pathway for photogenerated electron transport, which allows highly efficient photogenerated electron transport through the 3D dandelion-like structure and a lower *e*^−^/h^+^ recombination loss. For nanoparticles, when particle size becomes extremely small, i.e., several nanometers in diameter, surface recombination instead of volume recombination becomes a dominant regime. Most of the *e*^−^/h^+^ are generated sufficiently close to the surface. They may quickly reach the surface and undergo rapid surface recombination mainly due to abundant surface-trapping sites and the lack of driving force for *e*^−^/h^+^ separation. Another reason for the high photocatalytic activity of the sample prepared with 0.68 M HCl might be a mixed phase consisting of rutile and anatase types of TiO_2_ that prohibits the *e*^−^/h^+^ recombination [43]. For the heterogeneous anatase/rutile TiO_2_ system, photogenerated electrons are effectively accumulated in the rutile phase without recombining with the holes in the anatase valence band, resulting in the enhancement of the photocatalytic activity. Besides, less aggregation for the dandelion-like morphology facilitates higher photocatalytic activity. As expected, the nanorod fragments fabricated with 1.35 M HCl present lower photocatalytic degradation owing to the collapse of dandelion-like structure as well as severe aggregation and therefore lower surface area.

**Table 1:** Morphology, BET surface area, and rate constant of products synthesized with various acid conditions

**Acid condition**	**Unit morphology**^**a**^	**Size**^**b**^**(nm)**	**BET surface area (m**^**2**^**·g**^**−1**^**)**	**Rate constant*k*(min**^**−1**^**)**
0.68 M HCl	NR	13.5 ± 1.4	40.3	0.135
0.90 M HCl	NR	15.1 ± 1.3	38.1	0.095
1.12 M HCl	NR	17.8 ± 1.7	28.2	0.083
1.35 M HCl	NR	19.8 ± 1.8	18.7	0.074
0.90 M HNO_3_	NP	5.0 ± 0.5	190.9	0.104
0.90 M HAc	NP	8.2 ± 0.9	153.7	0.091

^a^The morphology of the unit in the hierarchical structure where NR and NP refer to nanorod and nanoparticle, respectively; ^b^the average diameter of the nanorods or nanoparticles estimated from the FESEM and TEM images.

## Conclusions

3D TiO_2_ hierarchical structures with various microstructures have been successfully synthesized by solvothermal method, in which HCl, HNO_3_, and HAc is used as the acid medium, respectively. The acid medium significantly affects the microstructures, optical properties, and photocatalytic activity of TiO_2_. The samples prepared with 0.68 to 1.12 M HCl possessed 3D dandelion-like spheres assembled of radially arranged rutile nanorods. Both the fraction of rutile and the diameter of nanorod are enhanced with the increasing HCl concentration. For the products derived from either HNO_3_ or HAc, 3D spheres composed of anatase nanoparticles were present. The unique 3D dandelion-like hierarchical structure exhibited low reflectance and efficient light harvesting since the ordered rods provided a light-transfer path for incident light and multiple reflective and scattering effects. 3D TiO_2_ prepared with 0.68 M HCl showed superior photocatalytic activity due to this unique topology and optical properties as well as anatase/rutile heterogeneous system. Considering the unique morphology of the 3D dandelion-like TiO_2_ structures, the recovery of these 3D TiO_2_ powders from the solution is believed to be much easier than that of nanoparticle powders.

## Competing interests

The authors declare that they have no competing interests.

## Authors' contributions

JZ participated in the design of the study, carried out the experiments, collected data, performed data analysis, and drafted the manuscript. GZ, BS, and GH participated in the design of the study, discussion of data, and helped to draft the manuscript.
